# Editorial: Endocrine Regulation of Insect Diapause

**DOI:** 10.3389/fphys.2022.962604

**Published:** 2022-06-30

**Authors:** Julie A. Reynolds, Wen Liu, Kunihiro Shiomi

**Affiliations:** ^1^ Department of Evolution, Ecology, and Organismal Biology, The Ohio State University, Columbus, OH, United States; ^2^ Hubei Key Laboratory of Insect Resources Utilization and Sustainable Pest Management, College of Plant Science and Technology, Huazhong Agricultural University, Wuhan, China; ^3^ Faculty of Textile Science and Technology, Shinshu University, Ueda, Japan

**Keywords:** circadian clock, juvenile hormone, aging, dormancy, developmental arrest

Insect diapause is an interesting puzzle that has captured the interest of researchers for many decades. It is an endogenously regulated dormancy that occurs in response to perceived changes in the environment (e.g., changes in the photoperiod or temperature) that signal the beginning of a season of adverse conditions. Perception of these changes leads to downstream effects including accumulation of fat reserves, developmental arrest, depressed metabolism, high resistance to stress, and increased lifespan. In between perception and response, the endocrine system has a role initiating the downstream changes associated with the diapause, however, the details of interactions between perception and response remain a significant knowledge gap. The aim of this Research Topic is to fill that gap by exploring processes related to the endocrine regulation of diapause in evolutionarily diverse insect taxa including Hemiptera, Lepidoptera, Coleoptera, and Diptera.

In their original research paper, Hara and Yamamoto describe a new method for evaluating the diapause status of a female fruit fly. Diapause in females of *Drosophila melanogaster* is characterized by the absence of yolk in the egg chambers of the ovary, a feature that is difficult to assess visually. Using a GFP fluorescent tag to label the major yolk protein, Yp1, the authors were able to more accurately measure changes in yolk protein and determine the diapause status of female *Drosophila*. The development of this new technique has the potential to facilitate future diapause research, and especially, development of the reproductive system.

A review by Hutfilz summarizes current knowledge about endocrine regulation of insect diapause in the context of aging and lifespan extension. The author discusses the roles of juvenile hormone, 20-hydroxyecdysone, prothoracicotropic hormone, adipokinetic hormone, and insulin in regulating diapause and, by extension, how they mediate the dramatic change in age progression that results from entering diapause. This summary illustrates how aging and diapause are related and contributes to our understanding of these overlapping fields.

Original research by Cambron-Kopco et al. shows that gene expression profiles of diapausing solitary bees, *Megachile rotundata*, depend on the timing of diapause entry and the environmental conditions experienced by overwintering pre-pupae. Specifically, they found differences in the mRNA expression of genes encoding transcription factors as well as several genes involved in insulin signaling, cell cycle regulation and cell growth. Together these results suggest that molecular regulation of diapause is a highly plastic response that depends on previous experience with the environment rather than a single, defined, shift in gene expression. The authors predict that these differences in gene expression indicate differences in diapause physiology, but they need to do more research to uncover those differences.

Two papers discuss how interactions between the circadian clock and the endocrine system regulate diapause. First, original research by Homma et al., examined how circadian clock genes regulate the temperature-dependent induction of diapause in *Bombyx mori*. Their study showed that knockout of the genes *period*, *timeless, clock, cycle,* and *cryptochrome 2* disrupts diapause induction at a point upstream of the GABAergic and diapause hormone signaling pathways. The second paper is a review by Takeda and Suzuki that considers four functional units of the photoperiodic system: a photo/thermo-sensitive input unit, a clock, a counter for tracking the number of signals perceived, and a neuroendocrine switch that initiates the phenotypic shift. The authors summarize the historical and current research on the photoperiodic system and circadian clock, and they discuss our current understanding of how integration of these units regulates diapause entry. Together these papers provide an interesting look at how the perception of a token cue (i.e., change in photoperiod or temperature) may lead to a physiological response.

The last two papers in this Research Topic look at the role of juvenile hormone in regulating diapause in two insect species that have economic importance. Zhou et al., report original research on the role of juvenile hormone in regulating adult, reproductive diapause in the pentatomid, *Aspongopus chinensis*. During diapause the level of juvenile hormone III (JHIII) is significantly lower than in post-diapause adults. Injecting exogenous JHIII restarts development of the reproductive system, increases mating activity, and increases feeding. Both JH biosynthesis and JH degradation pathways contribute to reproductive diapause in this bug. Finally, Li et al., report that an increase in the degradation of juvenile hormone regulates reproductive diapause in the ladybeetle, *Coccinella septempunctata*. Transcription of the JH degradation genes, *juvenile hormone esterase* and *juvenile hormone epoxide hydrolase* is elevated in diapausing females compared to their nondiapausing counterpart. Knockdown of these genes terminates diapause and promotes reproduction and reduces lipid accumulation.

Together these articles demonstrate that endocrine systems have a significant role translating environmental inputs to diapause outputs in insects, and they contribute to our understanding of the mechanisms involved, not only in diapause, but also in the phenotypic plasticity of animal development. In the long-term, these contributions have the potential to aid in improving the management of economically important insects. They also increase the usefulness of insect models for understanding human-related processes including obesity, metabolic regulation, and ageing.

